# In Situ Microfocus Chemical Computed Tomography of the Composition of a Single Catalyst Particle During Hydrogenation of Nitrobenzene in the Liquid Phase**

**DOI:** 10.1002/anie.201504227

**Published:** 2015-07-03

**Authors:** Stephen W T Price, Kalotina Geraki, Konstantin Ignatyev, Peter T Witte, Andrew M Beale, J Fred W Mosselmans

**Affiliations:** Science Division, Diamond Light Source, Harwell Science and Innovation Campus Didcot, Oxon, OX11 0DE (UK); UK Catalysis Hub, Research Complex at Harwell, Harwell Science and Innovation Campus Harwell, Didcot, Oxon, OX11 0FA (UK) University College London, Department of Chemistry 20 Gordon Street, London, WC1H 0AJ (UK) E-mail: andrew.beale@ucl.ac.uk; Catalysis Research GCC/PB BASF Nederland B.V. Strijkviertel 67, 3454 ZG, De Meern (The Netherlands)

**Keywords:** computed tomography, heterogeneous catalysis, liquid phase, supported catalysts, X-ray microspectroscopy

## Abstract

Heterogeneous catalysis performed in the liquid phase is an important type of catalytic process which is rarely studied in situ. Using microfocus X-ray fluorescence and X-ray diffraction computed tomography (μ-XRF-CT, μ-XRD-CT) in combination with X-ray absorption near-edge spectroscopy (XANES), we have determined the active state of a Mo-promoted Pt/C catalyst (NanoSelect) for the liquid-phase hydrogenation of nitrobenzene under standard operating conditions. First, μ-XRF-CT and μ-XRD-CT reveal the active state of Pt catalyst to be reduced, noncrystalline, and evenly dispersed across the support surface. Second, imaging of the Pt and Mo distribution reveals they are highly stable on the support and not prone to leaching during the reaction. This study demonstrates the ability of chemical computed tomography to image the nature and spatial distribution of catalysts under reaction conditions.

Recent developments in spectroscopy and diffraction imaging have allowed for the collection of spatially resolved chemical information (or chemical imaging) at the micrometer scale, combining computed tomography (CT) with techniques such as X-ray fluorescence (μ-XRF),[1] X-ray diffraction (μ-XRD),[1c, 2] and pair distribution function (PDF).[3] The high brilliance and monochromatic nature of synchrotron X-ray sources are ideal for measurements such as these on samples not only ex situ but also under reaction conditions. This has given valuable new information about the atomic-nanoscale chemical structure coupled with information about the micro-distribution of such species, with applications across a wide range of disciplines such as materials, biomedical, environmental, and geophysical sciences.[2a, 4] It is now possible to observe how different preparation methods or deposition sequences affect the micro-distribution of materials, and how structures respond to environmental changes, for example, changes in temperature, pressure, or gas treatments. In terms of catalysis, the formation, phase, and spatial distribution of the active species can be mapped from catalyst preparation[3, 5] right through to activation, operation,[2a],[d] and eventually to deactivation, consequently allowing for a better understanding regarding the correlation of structure and activity.

To date all previous imaging studies have focused on gas-phase catalytic systems, very few have been multimodal[1c, 6] and as yet, no chemical-CT studies have been performed on heterogeneous catalytic systems in the liquid phase. This is largely due to the challenges involved in generating real operating conditions whilst simultaneously collecting high-quality data. Indeed, although a large proportion of heterogeneous catalysis occurs in the liquid phase, the number of bulk in situ structural studies have been limited,[7] representing a significant gap in our understanding. Herein, we follow the hydrogenation of nitrobenzene to aniline in the liquid phase using a colloidal Pt/C catalyst promoted with Mo (NanoSelect). Traditional operating conditions for nitrobenzene hydrogenation have required either elevated temperatures[8] or pressures,[9] and more recent catalysts that operate under milder conditions have contained undesirable heavy metals such as Pb.[10] The advantage offered by the NanoSelect catalysts is that the operating conditions are much milder (30 °C and 4 bar H_2_, substrate:catalyst ratio of 20 000) and at the same time demonstrate improved performance,[10b] resulting in greatly reduced energy costs during the reaction. Our previous study on single catalyst particles revealed the chemical nature and distribution of both the Pt catalyst and the Mo promoter of the as-prepared catalyst, including details of the colloidal structure.[1b] Whilst the colloidal stabilizer is known to be removed during the reaction, it is not known what state the Pt and Mo are in under true operating conditions. Using a combination of X-ray absorption near-edge spectroscopy (μ-XANES), μ-XRF-CT, and μ-XRD-CT, we are able to determine what happens to the different Pt species, as well as to the Mo promoter, revealing the active state and stability of the catalyst through imaging a single catalyst particle. In situ chemical tomographic data collection was made possible by use of a specially designed microreactor. With the production of aniline alone generating in excess of 5 Mt year^−1^, an improved understanding of the structure and behavior of liquid-phase catalysts under operating conditions could lead to a better understanding into why this catalyst is both so active and selective. This information may eventually allow for significant improvements in efficiency in this and potentially other related catalytic systems.

In general, the catalyst particles have a Pt-rich shell with some Pt located in the core, and an even distribution of Mo throughout the core. The distribution herein remains one of Pt concentrated on the surface of the particle and Mo in the core (Figure 1, left; see also Figure S1 in the Supporting Information). A photograph of typical catalyst particles is included for comparison in Figure 1 (right). The location for the μ-XRF-CT slicing was chosen to cross through the particle where there was an obvious variation in the Mo distribution, approximately 60 μm from the bottom of the particle. The μ-XRF-CT slice under operating conditions (Figure 2; Figure S2) shows the Pt is almost entirely confined to the surface of the particle, with more than 90 % of the total Pt signal in the outer 5 μm layer. Inside this Pt shell lies the Mo, distributed throughout the internal pore structure of the carbon (see Movie S1 in the Supporting Information). Whilst there are some local variations in concentration of the two metals, the overall distribution under operating conditions is the same as that measured ex situ, indicating that no structural reorganization has occurred on a scale of 1 μm to hundreds of micrometers. The metal location would not necessarily be expected to change and this in situ measurement shows no evidence of serious leaching of the metals into solution.

**Figure 1:**
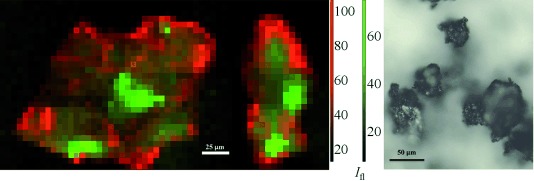
Left: Orthogonal XRF maps (at 20.3 keV) of a particle under operating conditions (ethanol, 22.3 mm nitrobenzene, and 1 bar H_2_) after collection of μ-XRF-CT and μ-XRD-CT sinograms. Elemental distributions throughout the carbon support are shown in red (Pt) and green (Mo). Each pixel is 5×5 μm. Red and green scale bars at the side show fluorescent signal intensity (*I*_fl_), white scale bar (in the image)=25 µm. Right: Photo of typical catalyst particles (ex situ); scale bar=50 μm.

The results of our previous study revealed a colloidal structure consistent with Cl exchange of the phosphate anion of hexadecyl(2-hydroxyethyl)dimethylammonium dihydrogenphosphate (HHDMA) resulting in the formation of Pt nanoparticles surrounded by an inner Cl shell and an outer HHDMA shell, resulting in the distinctive Pt XANES spectrum.[1b] Once under operating conditions, the Pt XANES spectrum changes (Figure S3) to a form consistent with reduced Pt species. Indeed linear combination fitting (LCF) of the XANES confirms that only Pt metal is present, with none of the oxide or chloride near-neighbor contributions previously observed (Figure S4).[1b] The HHDMA shell is known to be soluble in ethanol,[11] and so is lost on immersion of the catalyst in the reaction solvent, along with the Cl shell. This is responsible for the reduction of the Pt catalyst (detected in the XANES), rather than any reduction by the H_2_ present. This correlates well with previous carbon analysis of similar colloidal Pd–TiS catalysts, whereby the deliberate removal of the stabilizing HHDMA shell prior to hydrogenation, by washing with ethanol, did not affect the catalytic activity.[12] It is likely however that the hydrogen environment of the reaction prevents the passivation of the surface of the nanoparticles.

Whilst the data collected for each μ-XRF-CT slice contained sufficient information for a smooth reconstruction (Figure 2; Figure S2), the μ-XRD-CT measured at the same location contains far less information. The colloidal preparation method results in a very well prepared catalyst with only a minor amount of crystalline Pt (or other phases) present. The scarcity of crystalline Pt means that a clear sinogram feature cannot be formed, giving rise to problems during reconstruction; the exact location in real space of the diffracting feature cannot be accurately resolved (Figure S8). We know, however, that the feature is present at some point along the X-ray path through the sample and so by overlaying the XRF and XRD reconstructions, the approximate location of the crystalline Pt can be determined from the point of intersection of the Pt(111) reflection and the Pt XRF signal (Figure 2). The XRD pattern, summed over all the pixels in the image and corresponding to the XRF-CT slice from Figure 2, is shown in Figure S9. It is worth noting that the scarcity of Pt(111) reflections across the surface of the particle is indicative of the nature of the surface Pt species. Specifically, the colloidal preparation method finely controls the size (and dispersion) of the particles so the average particle size is below 1 nm with the vast majority either atomic or clustered, a size which is too small to coherently diffract, as well as ensuring minimal larger crystals.[1b] Once the HHDMA is removed, the particles are sufficiently dispersed on the carbon so as to not agglomerate (and crystallize), with the majority of Pt atoms highly undersaturated, providing a large number of active sites for catalysis. The few crystallites detected by the μ-XRD-CT may be a result of a localized concentration of colloid during the deposition stage that aggregated into single large particles once the HHDMA colloid was removed. Performing a Scherrer analysis, we estimate that the few particles that do diffract (Figure S9) have an estimated particle size between 50 and 70 nm. Very similar metal distributions were detected for all other μ-XRF-CT slices measured through the particles under operating conditions (Figure S2). However, the corresponding μ-XRD-CT slices contained no Pt(111) reflections. There was no noticeable diffraction from the Mo complex in the regions measured during the reaction.

**Figure 2:**
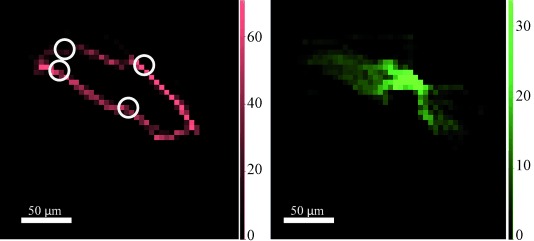
Cross-sections from μ-XRF-CT at 20.3 keV showing Pt (red, left) and Mo (green, right) distributions throughout the carbon support. Each pixel is 5×5 μm. Data collected under operating conditions (ethanol, 22.3 mm nitrobenzene, and 1 bar H_2_). The slice corresponds to a horizontal cross-section 60 μm from the bottom of the particle (see Figure 1). The white circles correspond to the approximate locations of the Pt crystals (identified from the 111 reflection). Scale bars at the sides show fluorescent signal intensity (*I*_fl_), white scale bars (within the images)=50 µm.

Given that a few Pt crystallites were detected on the surface of the single particle, it follows that an in situ bulk XRD measurements on a sample (consisting of more than 10^6^ particles) under operating conditions would reveal a clear Pt diffraction pattern. From such an XRD measurement, it may be falsely concluded that the catalyst crystallizes under the reaction conditions and therefore the active form of the Pt catalyst is nanocrystalline. The advantage offered by single-particle μ-XRD-CT is that whilst a small amount of crystalline Pt is detected, the vast majority of Pt species, determined in combination with μ-XRF-CT, is too small to diffract coherently, and therefore alters the conclusion such that the active catalyst is not crystalline nanoparticles but Pt nanoclusters.

The catalysis was confirmed using Raman spectroscopy. The spectrum measured immediately after the tomography collection (Figure 3) shows a split band for a ring-breathing mode of the phenyl ring. This particular mode was chosen as it is the only clear reactant/product band visible at the given reaction concentration. The peak at higher wavenumber (1003.6 cm^−1^) is that of the nitrobenzene precursor, and that at lower wavenumber (998.2 cm^−1^) corresponds to aniline. The presence of both peaks confirms: a) that the catalyst was converting nitrobenzene into aniline, and b) that the reaction had been slowed sufficiently so as not to have completed before the CT data was collected, meaning that all μ-XRF-CT, μ-XRD-CT, and μ-XANES measurements were collected whilst the catalyst was in its active state. It may be that some hydroxylamine intermediate is also present in the capillary given that the reaction had not completed, however the reaction spectrum can be replicated by mixing aniline with nitrobenzene in a 3:1 ratio, suggesting that conversion was over 75 % complete at the time of finishing the CT measurements (see also Figure S10). Although the spectra in Figure 3 are noisy, it should be noted that these spectra are recorded on less than 1 μL of solution within the microreactor.

**Figure 3:**
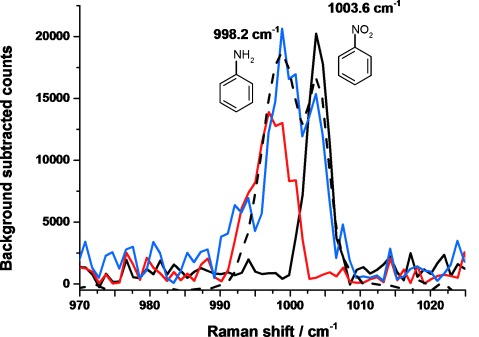
Raman spectra (*λ*_exc_=785 nm) of reference solutions of the reactant nitrobenzene (22.3 mm in ethanol; black trace), the product aniline (22.3 mm in ethanol; red trace), and of the reaction solution in the capillary at the end of data collection (blue trace). A spectrum of an aniline/nitrobenzene mixture (3:1 ratio; black dashed trace) is included to provide an estimate of the extent of the conversion at the end of imaging.

Herein we have reported the first instance of the imaging of the elemental and phase distribution of an industrial catalyst during liquid-phase hydrogenation reaction conditions using a combination of μ-XRF-CT and μ-XRD-CT. In this particular study XRF revealed that the elemental distribution during the conversion of nitrobenzene into aniline does not change under operating conditions, and that the total fluorescence intensity remains constant indicating no loss of Pt from the surface of the support. Additional μ-XANES measurements confirm the loss of the stabilizing layer (and the associated Cl inner shell), generating highly undercoordinated nanoclusters of Pt on the carbon support. Importantly, the Cl ions, once freed from the colloid shell, do not interfere with the hydrogenation, as shown by the catalytic data.[10b, 11, 12] The μ-XRD-CT reveals the vast majority of the Pt catalyst to be noncrystalline, with only a few Pt crystals on the surface of the support, confirming the effectiveness of the colloidal preparation method (minor imperfections in the preparation process result in the few crystallites observed). The very small size of the Pt and Mo species is in agreement our previous ex situ μ-XANES-CT study where no crystallites were detected.[1b] Although the support loadings of individual particles has been shown to vary,[13] the elemental distributions are consistent across all four particles measured in this and our previous study and, the particles chosen are representative of the sample. This study highlights the importance of imaging catalysts on different length scales as the very high proportion of noncrystalline catalyst would not have been apparent if imaged on a larger length scale, such as a pellet with tens of micrometer resolution.

## Experimental Section

The fresh cPt+Mo/C catalyst (1.6 wt % Pt, 0.6 wt % Mo) was supplied by BASF and used as received. A higher loading was used to improve the signal to noise ratio; the catalyst preparation procedure however was the same as has been detailed previously.[10b, 11] A single particle was mounted in 0.4 mm OD borosilicate glass capillary (0.01 mm wall thickness) using quartz wool and H_2_ purged ethanol was injected into the capillary (circa 10 μL) then pressurized to 1 bar with H_2_. For the measurements under operating conditions, a second capillary was prepared with the addition of nitrobenzene (22.3 mm, 43 nL ≥99.0 % Sigma Aldrich) in 10 μL ethanol. The capillary was sealed and mounted on top of two Attocube nanopositioning stages to allow for centering of the particle on the axis of rotation (Figure S11), forming the capillary microreactor. Under optimum conditions (30 °C and 4 bar H_2_, 1500 rpm stirring)[10b, 11, 12] the conversion into aniline is very fast (ca. 1 h for full conversion) with more than 99 % selectivity (selectivity and activity were determined by GC–MS when the uptake of hydrogen ceased[10b]). However, the activity was moderated (23 °C and 1 bar H_2_, with no stirring because of the capillary dimensions) to suit the conditions for performing tomography so as to allow for real-time imaging during steady-state catalytic turnover. In all particles chosen in this and in our previous study,[1b] the elemental distribution is consistent, and does not appear to be influenced by particle size. It should be noted that with the lack of stirring, diffusion becomes the main mass-transport process of reactant/product and therefore may influence the activity of the catalyst, and also the ratio of products detected by Raman spectroscopy.

XANES data were recorded at the Pt L_3_ absorption edge (11 564 eV) at beamline I18 of the Diamond Light Source operating with a Si(111) double crystal monochromator, with the X-ray beam focused to a 2×2 μm (FWHM) spot on the sample using Kirkpatrick–Baez mirrors. Data was collected in fluorescence mode using a 9-element Ge solid-state detector and XSPRESS-2 electronics.[14] Calibration of the monochromator was performed using a Pt foil measured in transmission mode. The X-ray absorption spectroscopic (XAS) data were processed using Athena.[15] LCF of the XANES was carried out as previously reported.[1b]

XRF-CT data were collected using an incident X-ray energy of 20 300 eV with a spot size of 2×2 μm (FWHM). The sample was rastered across the beam in a translate–rotate data collection scheme, similar to 1st generation CT with a pencil beam, with an XRF spectrum collected at 5 μm intervals with a collection time of 0.3 s/pixel. Typically a total of 41 translation steps at each rotation angle and 37 rotations were used (exact number of translations was dependent on particle width). The regions of interest from the XRF sinograms were extracted and reconstructed as previously reported.[1b]

XRD-CT data were collected in the same manner as with the XRF-CT data but with a 13 000 eV incident X-ray beam, and with a collection time of 2.0 s/image. The images were recorded with a Photonic Sciences CMOS-based X-ray imaging detector. The detector was calibrated (using a LaB_6_ reference material), and data azimuthally integrated from 2D images to 1D patterns using Dawn v1.7.[16] Each 2*θ* step in the reduced dataset was then filter-back-projected to create the final 3D datastack; having 2 spatial axes (*x*, *y*) and the 3^rd^ axis (*z*) being the reconstructed pattern (2*θ*). Each pair of XRF-CT and XRD-CT measurements took 2 h; the measurements under operating conditions were recorded over a circa 24 h period.

Raman spectroscopy was collected using a Renishaw InVia microscope with a *λ*=785 nm laser. Spectra were measured of the solution in the capillary before exposure to H_2_, and after the collection of the tomographic data (i.e. after exposure to H_2_). A spectrum was also measured of a reference solution of aniline (22.3 mm) in ethanol, corresponding to the expected complete conversion product.

## Supporting Information

As a service to our authors and readers, this journal provides supporting information supplied by the authors. Such materials are peer reviewed and may be re-organized for online delivery, but are not copy-edited or typeset. Technical support issues arising from supporting information (other than missing files) should be addressed to the authors.

miscellaneous_information

movie_1_ptmo
