# Supplementary material for: In Situ Microfocus Chemical Computed Tomography of the Composition of a Single Catalyst Particle During Hydrogenation of Nitrobenzene in the Liquid Phase
Source: Angew Chem Int Ed Engl. 2015 Jul 3;54(34):9886–9. doi: 10.1002/anie.201504227 (PMC4600245; doi:10.1002/anie.201504227)
Supplement: Supplementary file 1 — miscellaneous_information [file anie0054-9886-sd1.pdf]

Angewandte Chemie
Eine Zeitschrift der Gesellschaft Deutscher Chemiker

Supporting Information

In Situ Microfocus Chemical Computed Tomography of the Composition of a Single Catalyst Particle During Hydrogenation of Nitrobenzene in the Liquid Phase**

Stephen W. T. Price, Kalotina Geraki, Konstantin Ignatyev, Peter T. Witte, Andrew M. Beale,* and J. Fred W. Mosselmans*

anie_201504227_sm_miscellaneous_information.pdf
anie_201504227_sm_Movie_1_PtMo.wmv

Supplementary Information

SI Movie 1: Reconstruction of *ex situ* XRF-CT of cPt+Mo/C sample. Pt distribution in red, Mo distribution in green. Collected at 20.3 keV incident X-ray energy. Particle is ca, 35x40x80 µm.

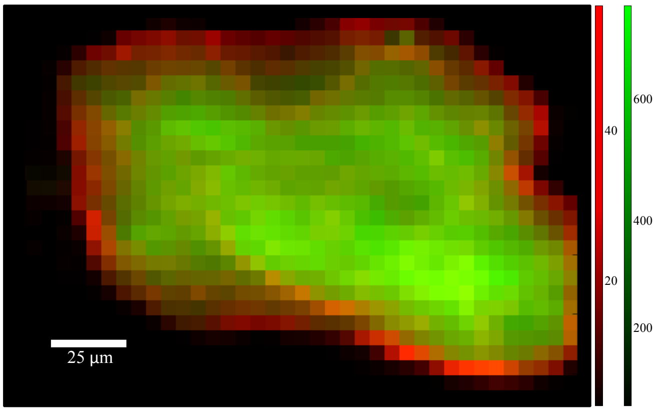

SI Figure 1: µ-XRF map at 20.3 keV: Pt (red) and Mo (green) distributions throughout the carbon support. Each pixel is 5 x 5 µm. Data collected under *in situ* conditions (ethanol and 1 bar H_2) but without presence of nitrobenzene. Scale bars represent fluorescent signal intensity (au).

XRF mapping of particles *in situ* (SI Fig 1) and during operation (Fig 1) reveals more inhomogeneity in the element distributions, in particular with Mo, than in previously mapped particles[1]. The Mo signal from the core of the particle measured *in situ* is far stronger than that for the particle measured under operating conditions, and also far higher than that measured for the particles *ex situ*[1], indicating there is a greater degree of variation in the concentration of Mo deposited than for Pt. It may be that in this instance the lower surface coverage of the Pt colloid allowed for a far greater amount of Mo to reach the inner pore structure of the carbon. The greater Mo variation observed may be a result of the doubled loadings of catalyst and promoter, chosen to improve the signal to noise ratio.

The intensities of each elemental signal in the XRF map (Fig 1) are higher than those in the reconstructed sinograms (Fig 2 and SI Fig 2). However, the intensities of each pixel of the XRF map are the sum of all the counts through the particle at the given projection whilst the sinograms are spatially resolved. The intensities of each element vary slightly between sinograms, as can be seen from the map (Fig 1), this is the result of small variations in the deposited concentration throughout the support.

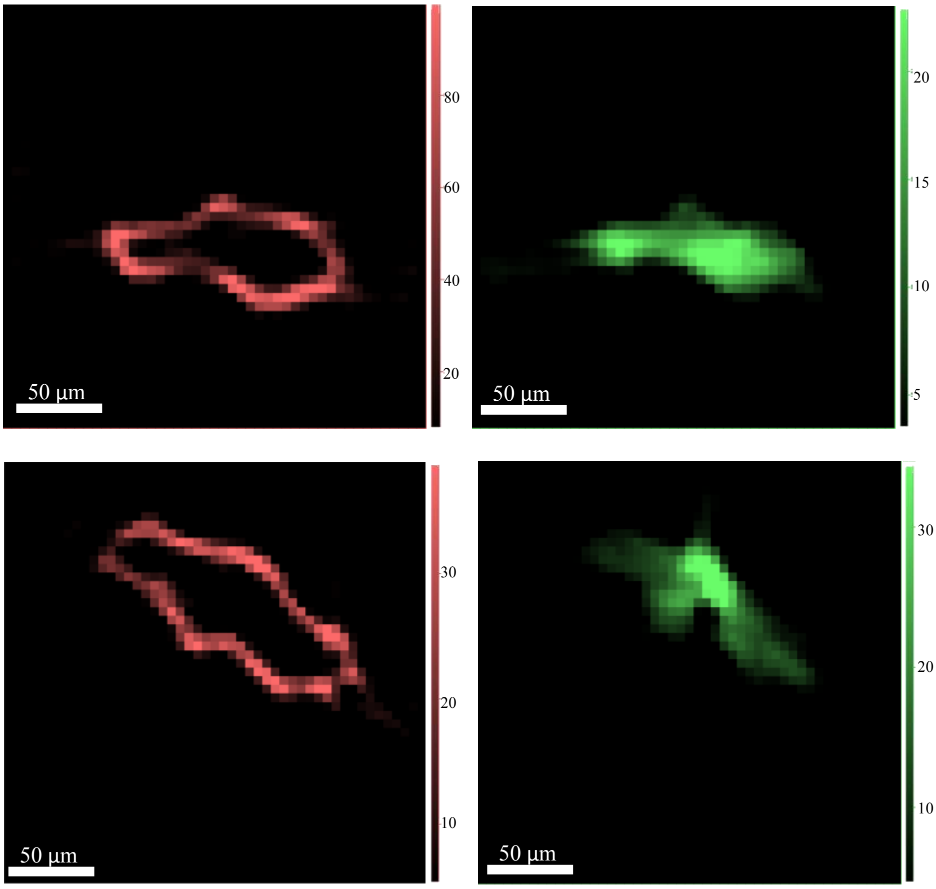

SI Figure 2: Horizontal cross-sections from μ-XRF CT at 20.3 keV: Pt (left) and Mo (right) distributions throughout the carbon support. Each pixel is 5 x 5 μm. Data collected under operating conditions (ethanol, 22.3 mM nitrobenzene and 1 bar H_2). Corresponding diffraction data was collected at these orientations however no diffraction peaks were present. Scale bars represent fluorescent signal intensity (au).

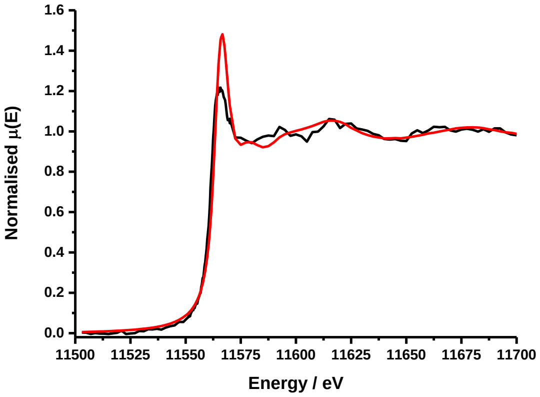

SI Figure 3: Pt L_3 edge XANES spectra of cPt + Mo/C catalyst *ex situ* (pressed pellet) (red) and under operating conditions (single grain) (black); ethanol, 1 bar H_2 and 22.3 mM nitrobenzene.

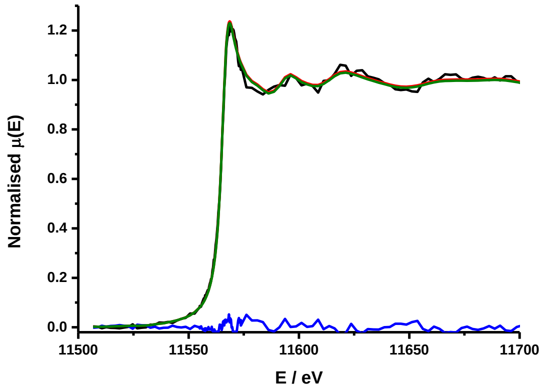

SI Figure 4: Results of LCF of Pt L_3 edge XANES under operating conditions (ethanol, 22.3 mM nitrobenzene and 1 bar H_2); experimental data (black), fit (red), residual (blue) and Pt/C nanoparticle reference (green). The R-factor of the linear combination fit in Athena is 0.0016 (measured between 0 and 1 where 0 equates to a perfect fit and 1 failure to fit), and the majority of the residual is attributed to experimental noise. Every other reference spectra fitted with a contribution of less than 1% with an error at least double the contribution.

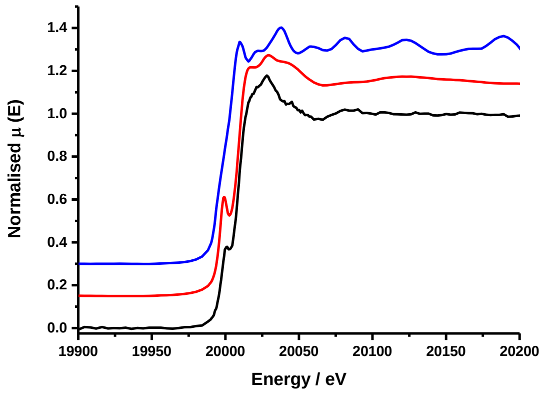

SI Figure 5: Mo K edge XANES spectra of Mo Foil (blue), cPt + Mo/C catalyst *ex situ* (pressed pellet -red) and under operating conditions (single grain - black); ethanol, 1 bar H_2 and 22.3 mM nitrobenzene.

The Mo XANES under operating conditions differs from that *ex situ* (which corresponds to the heptamolybdate complex), and is likely a result of both coordination environment and oxidation state changing (SI Fig 5). The spectral shape over the absorption edge changes, and it is likely that this is a reduction of the molybdate[2] from $[Mo_7O_{24}]^{6-}$ to Mo_4O_{11}. The switch from all octahedral sites to a combination of tetrahedral and octahedral sites in Mo_4O_{11} would explain both the reduction in the intensity of the pre-edge feature as well as the increase in the rising edge intensity[3].

The molybdate may have been partially reduced by the hydrogen in the reaction, although the temperature of the reactor is far lower than would normally be used and as such partial reduction of the sample by the X-ray beam must be considered. This was observed at the Pt L_3 edge for the sample *ex situ*[1], and a small degree of beam interaction is also observed under operating conditions (SI Fig 6).

The very low volume of ethanol caused complications during the data collection as localised heating occurred due to high flux of the X-ray beam, leading to micro-bubbles forming in the capillary and necessitating reducing the exposure time of the solution to the X-ray beam. Whilst the movement of these bubbles within the capillary may have helped to circulate the reaction solution (since stirring was not possible), the extent of this is likely to have been limited; thus it is unlikely that the final solution in the capillary was thoroughly mixed, and therefore quantification of the turnover of nitrobenzene to aniline is not possible.

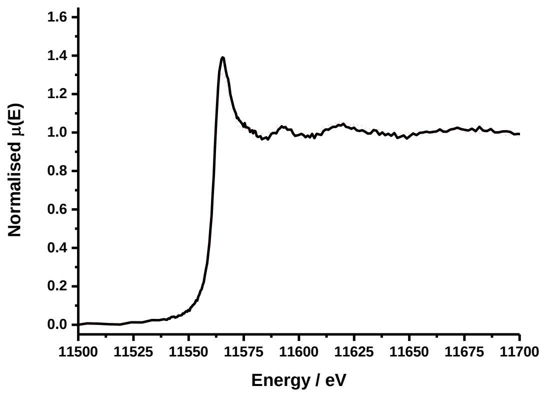

SI Figure 6:   Reduction by beam Pt XANES – as particles are already very small may be a result of decrease in particle size (or beam damage). First scan (black), second scan (red).

The degree of reduction is far less than that observed for the sample *ex situ* as the oxidising colloid shell has already been removed. Both the increased absorption edge intensity and the change in the immediate post edge feature between 11570-11575 eV are indicative of an increase in Pt nanoparticle size. The X-ray beam may be increasing the mobility of the Pt clusters on the surface of the carbon, leading to localised aggregation of the clusters that, whilst still too small to be observed by XRD, is enough to cause a change in the XANES due to an increase in the degree of multiple scattering of the photoelectron.

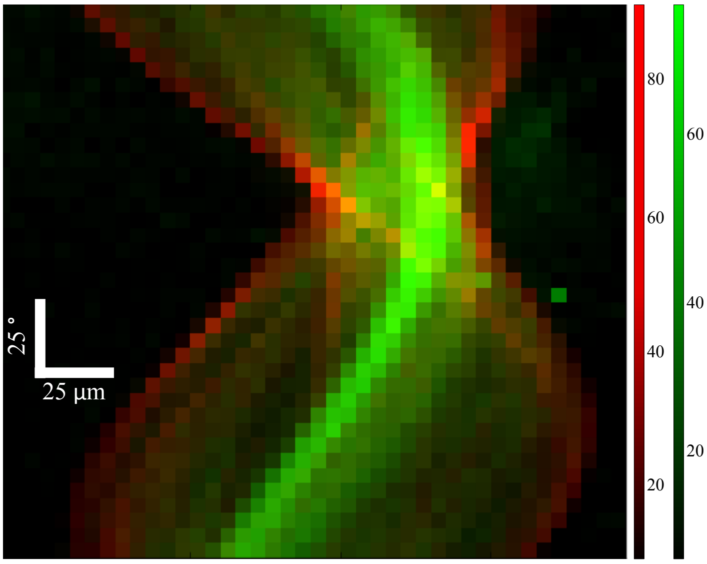

SI Figure 7: XRF-CT sinogram with 5 µm translation steps and 5 ° rotation steps, collected under operating conditions corresponding to reconstructions in Figure 5. Pt distribution in red, Mo in green. Scale bars represent fluorescent signal intensity (au). Corresponding XRD-CT sinogram is SI Fig 8.

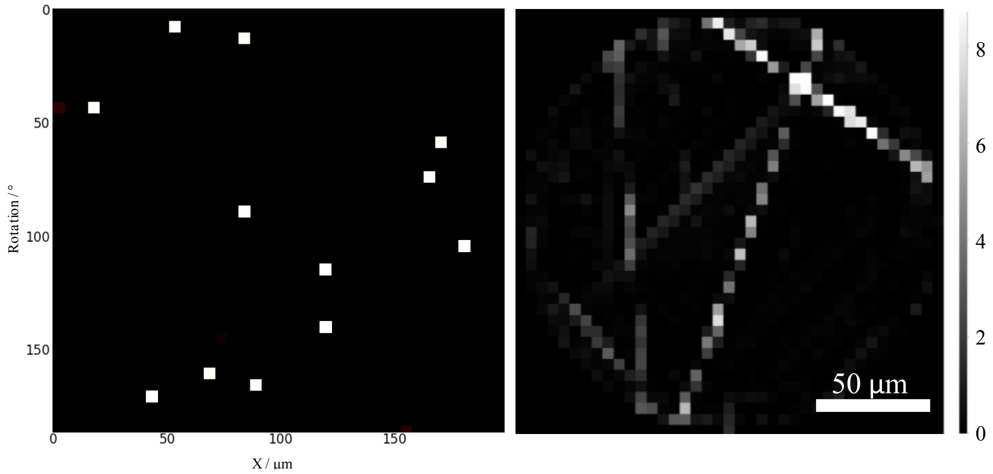

SI Figure 8: Left - Sinogram from µ-XRD-CT at 13.0 keV showing Pt 111 reflections only (23.7 - 24.5 ° 2θ); corresponds to XRF slices in Figure 5. Each pixel is 5° x 5 µm. Right – filter back projected reconstruction of the Pt 111 reflection sinogram. Corresponding XRF-CT sinogram is SI Fig 7.

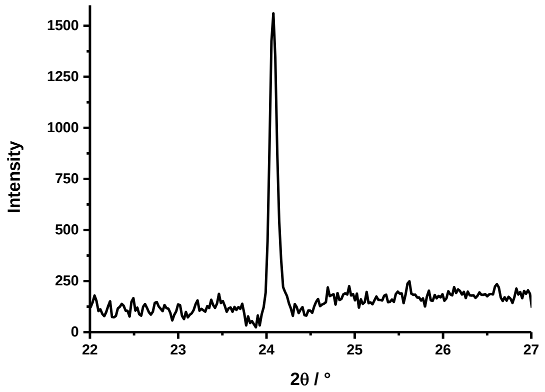

SI Figure 9: Background subtracted summed pattern from μ-XRD-CT slice (SI Fig 8) corresponding to μ-XRF-CT in Fig 2. Peak plotted is Pt 111 reflection. The Pt 111 diffraction peak is at 24.1° 2θ due to the energy of the incident X-ray beam (13.0 keV); the d-spacing associated with the reflection, 2.282 Å, is consistent with crystalline Pt.

Each row in the sinogram is a projection through the particle; a diffraction peak occurring at a certain point in the row is indicative of crystalline material being present at some point along the path of the X-ray beam through the sample. Normally when collecting XRD-CT on (poly)crystalline samples, a feature can be tracked through all rotations, forming the distinctive wave through the sinogram. However if only a few crystallites are present, then diffraction will only occur at a discrete number of rotations. The algorithms underlying the tomographic reconstruction rely on a continuous "wave" of data through the sinogram, and so fail to cleanly reconstruct the limited amount of information. The streak artefacts in SI Fig 8 are an example of reconstruction with very low information content in the sinogram.

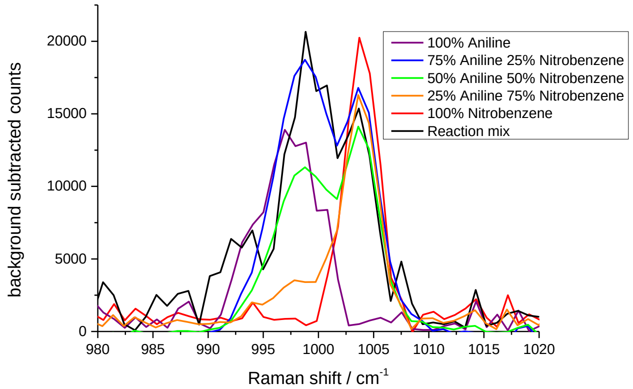

SI Figure 10: 785 nm Raman spectra of 22.3M reference solutions of aniline and nitrobenzene in ethanol, with mixtures of 25, 50 and 75% nitrobenzene with aniline (also in ethanol). The 75% aniline/25% nitrobenzene ratio best reproduces the peak shape observed for the reaction mixture. All spectra were measured in identical capillaries to that used for the tomography measurements.

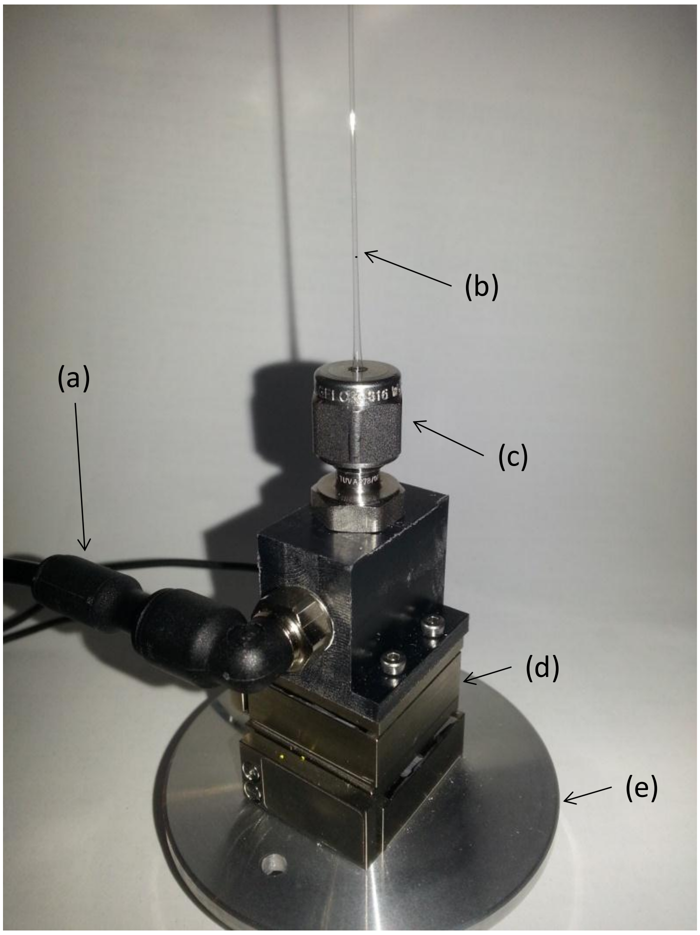

SI Figure 11: Microreactor for in situ tomography. (a) gas inlet, (b) single catalyst particle mounted in 0.4 mm diameter glass capillary, (c) capillary sealed with two o-rings, (d) nano-positioning motors for centring capillary, (e) base to connect to beamline rotation stage.

References

[1] S. W. T. Price, K. Ignatyev, K. Geraki, M. Basham, J. Filik, N. T. Vo, P. T. Witte, A. M. Beale, J. F. W. Mosselmans, *Physical Chemistry Chemical Physics* **2015**, *17*, 521-529.
[2] T. Ressler, J. Wienold, R. E. Jentoft, T. Neisius, *Journal of Catalysis* **2002**, *210*, 67-83.
[3] H.-K. Fun, P. Yang, M. Sasaki, M. Inoue, H. Kadomatsu, *Powder Diffraction* **1999**, *14*, 284-288.
